# Feasibility of the “Bring Your Own Device” Model in Clinical Research: Results from a Randomized Controlled Pilot Study of a Mobile Patient Engagement Tool

**DOI:** 10.7759/cureus.535

**Published:** 2016-03-16

**Authors:** Laura Pugliese, Molly Woodriff, Olga Crowley, Vivian Lam, Jeremy Sohn, Scott Bradley

**Affiliations:** 1 Innovation Research, HITLAB; 2 CentrosHealth founder, Clinical Ink; 3 Strategy and Operations, Clinical Ink

**Keywords:** mhealth, patient engagement, clinical trial tools, medication adherence, byod

## Abstract

**Background:**

Rising rates of smartphone ownership highlight opportunities for improved mobile application usage in clinical trials. While current methods call for device provisioning, the "bring your own device” (BYOD) model permits participants to use personal phones allowing for improved patient engagement and lowered operational costs. However, more evidence is needed to demonstrate the BYOD model’s feasibility in research settings.

**Objective:**

To assess if CentrosHealth, a mobile application designed to support trial compliance, produces different outcomes in medication adherence and application engagement when distributed through study-provisioned devices compared to the BYOD model.

**Methods:**

87 participants were randomly selected to use the mobile application or no intervention for a 28-day pilot study at a 2:1 randomization ratio (2 intervention: 1 control) and asked to consume a twice-daily probiotic supplement. The application users were further randomized into two groups: receiving the application on a personal "BYOD” or study-provided smartphone. In-depth interviews were performed in a randomly-selected subset of the intervention group (five BYOD and five study-provided smartphone users).

**Results:**

The BYOD subgroup showed significantly greater engagement than study-provided phone users, as shown by higher application use frequency and duration over the study period. The BYOD subgroup also demonstrated a significant effect of engagement on medication adherence for number of application sessions (unstandardized regression coefficient beta=0.0006, p=0.02) and time spent therein (beta=0.00001, p=0.03). Study-provided phone users showed higher initial adherence rates, but greater decline (5.7%) than BYOD users (0.9%) over the study period. In-depth interviews revealed that participants preferred the BYOD model over using study-provided devices.

**Conclusions:**

Results indicate that the BYOD model is feasible in health research settings and improves participant experience, calling for further BYOD model validity assessment. Although group differences in medication adherence decline were insignificant, the greater trend of decline in provisioned device users warrants further investigation to determine if trends reach significance over time. Significantly higher application engagement rates and effect of engagement on medication adherence in the BYOD subgroup similarly imply that greater application engagement may correlate to better medication adherence over time.

## Introduction

The pervasiveness of personal smartphone ownership provides new opportunities to rethink traditional clinical trial methodological models [1-2]. Mobile technology allows study teams to reach clinical trial patients directly on their personal phones through mobile applications (or “apps”) for study-related communications, data collection, and patient engagement—bringing the potential for applications to serve as a medium for improved patient management and retention [3-4]. Moreover, mobile phones offer the advantage of real-time communication, high usability, affordability, and ubiquitous access. Though standard methodological practice in clinical research utilizing smartphone applications and collecting electronic patient-reported outcomes (ePRO) has been used to provide participants with designated study devices, certain limitations constrain such methods [5]. One potentially promising alternative for improving patient engagement in clinical trial settings is the “bring your own device” (BYOD) model, which allows patients to use their personal internet-enabled smartphones in clinical trial research.

Provisioning devices is widely used in clinical research, but can be inconvenient for study subjects and costly to researchers, as research staff and participants must be trained on device usage, maintenance, and technical support in addition to the costs of provisioned devices [6]. Allowing patients to use their personal smartphones in clinical research may thus help to alleviate cost and logistical burdens on researchers, streamline data collection processes, and enhance convenience for patients, positively impacting patient engagement. Furthermore, with the potential for improved patient experience in clinical trials with BYOD, we may see positive gains as well in patient compliance and trial retention rates—major factors in clinical trial success and data validity. 

The BYOD movement originally gained momentum in higher education settings and in corporate workplaces as increasing numbers of organizations have been allowing employees to use their own smartphones and tablets at work, with the hope of improving employee engagement and reducing employer costs [7-8]. BYOD has since become a compelling model in the healthcare industry, and in clinical trial research particularly, for similar reasons: its potential to promote deeper patient engagement and reduce operational costs [5,8].

Despite the potential for the BYOD model to improve patient engagement and clinical trial processes, to our knowledge there have been no randomized controlled studies examining its feasibility for clinical trial research. This randomized controlled study aims to assess the differences in medication adherence and engagement among BYOD users and study-provided device users of a mobile application designed to improve participant medication and application engagement. In this way, we aim to elucidate best practices in mobile patient engagement in a clinical trial setting.

## Materials and methods

### Study design

A two-arm randomized, controlled, 28-day pilot study was conducted with participants (N=87) randomized to use either (1) the mobile application or (2) no treatment. Both groups were instructed to follow a twice-daily probiotic treatment regimen. The intervention arm was divided into two subgroups, where participants were randomly selected using an unblinded simple randomization schema to receive the mobile application on either (1) their personal smartphone (henceforth referred to as BYOD) or (2) a study-provided smartphone. Randomization was set at a 2:1 ratio with two subjects randomized to the intervention to every subject randomized to the control group. This paper assesses results from the intervention subgroups only. The primary endpoint, medication adherence, was indicated by pill count collected at Day 14 (+/- 2 days) and Day 28 (+/- 2 days) in person by trained researchers. Application usage data was collected and analyzed for differences between intervention subgroups. A nested qualitative assessment consisting of semi-structured interviews was also performed in a randomly selected subset of 10 participants from the intervention group (n=5 from the BYOD subgroup; n=5 from the study-provided phone subgroup) to assess the usability and acceptability of the mobile intervention across both modes of delivery. 

### Study population and recruitment

Participants local to the New York Metropolitan area were recruited from August-October 2014 through posted advertisements on online forums for patient recruitment and through paper flyers displayed at local universities and public spaces, inviting participation in a research study. The advertisement provided a description of the study and instructed interested persons to be pre-screened by phone to determine eligibility. The eligibility criteria were as follows: aged ≥ 18 years; in possession of an iOS-enabled smartphone device (iPhone) with internet, messaging capabilities, and iPhone App Store access; self-reported dissatisfaction with digestive health, as indicated by a score of four or below on a Likert scale assessment; willing to use the mobile application on personal iPhone or study-provided iPhone, and to complete required assessment visits; not using fiber supplements, probiotics, or prebiotics within the past thirty days; not immunocompromised or immunosuppressed; not allergic to soy; not pregnant or nursing (lactating); able to comprehend spoken or written English, write in English or read/respond to English text messages on smartphone; able to understand and provide written informed consent and to understand all study procedures; willing to comply with all study requirements.

### Intervention

CentrosHealth is a HIPAA-complaint smartphone application designed for use by participants in clinical trials to maximize participants’ protocol compliance and engagement in the clinical trial. It provides participants with personalized reminders about medication, treatment, site visits or other study-related activities, educational content related to the study, and contact information for study implementers. The application permits participants to log completed medication dosages, visible to researchers, and track their medication adherence history. The application also permits study data collection through questionnaires.

### Quantitative methods

The primary study endpoint was medication adherence as measured by pill count, defined as number of pills present at visit/number of pills expected at visit and analyzed using a mixed model approach. The mixed model approach has greater power compared to more traditional methods like analysis of variance (ANOVA), as it handles missing data more effectively and is more efficient, parsimonious, and flexible [9]. Separate models were tested for the two dependent variables: (1) the number of sessions and (2) the amount of time spent in each application session. The Bonferroni adjustment was used for each variable to control for Type I error (0.05/2 = 0.025). Covariates added to the analysis included participants’ age, gender, race, education, income, and number of diseases and medications (computed as a total count). Analyses, performed using SAS 9.3 (SAS Institute, Cary, North Carolina, USA), were adjusted for imbalances in the study groups and study population at baseline.

Application usage data was collected through Google Analytics from both the BYOD and study-provided mobile application subgroups, including the duration and frequency of application sessions. In the analysis of usage data across the two subgroups, a preliminary test for the equality of variances (F-test) was performed to determine whether the two groups had equal or unequal variances. Subsequently, a two-sample unpaired t-test was performed assuming either equal or unequal variances, depending on the outcome of the F-test. The significance level was 0.05. The Bonferroni adjustment was used to control for Type I error (0.05/2 = 0.025).

An additional analysis was performed to determine whether rates of engagement with the mobile application affected medication adherence in the mobile intervention arm and in the study phone and BYOD subgroups. Engagement was measured as (1) the number of sessions and (2) the amount of time spent in each application session, and was tested to see if it mediated the effect of the mobile intervention on medication adherence. In so doing, we used the Baron and Kenny (1984) approach to mediation analysis [10].

### Qualitative methods

Five individuals from each mobile application subgroup were randomly selected to voluntarily participate in 30-minute semi-structured interviews to assess user perceptions and experiences of using the mobile application. Differences in experiences and reported usability of the application were assessed across the BYOD and study-provided subgroups. A systematic analysis of the qualitative data was conducted to determine the usability of and user satisfaction with the mobile application. Analysis started with an a priori list of codes, based on the contents of the qualitative interview guide, with additional codes added during the coding process to allow for emergence of important themes from the data. Analysis was performed by staff trained in qualitative research methods using Atlas.ti version 6.2.

### Ethical approval

This study was conducted in accordance with the ethical principles originating in the Declaration of Helsinki and consistent with Good Clinical Practice and applicable regulatory requirements. The study was performed in accordance as well with the regulations of the United States Food and Drug Administration (FDA) as described in 21 CFR 50 and 56, applicable laws, and the standards and guidelines outlined by the Chesapeake IRB. Written informed consent was obtained from all subjects prior to participating in this study.

## Results

**Figure 1:**
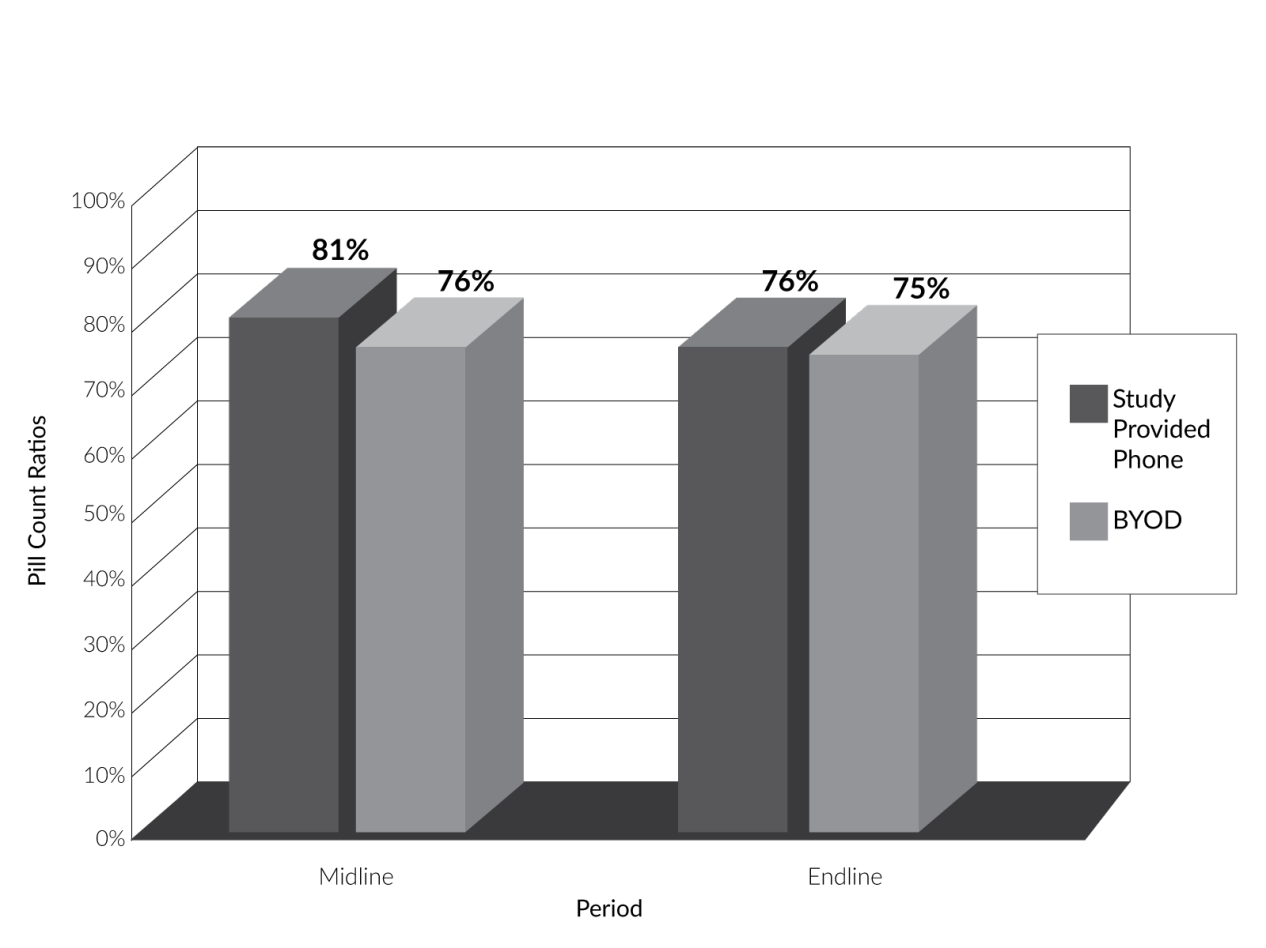
Pill count ratio by study group and period

Study-provided phone users and BYOD users did not have a statistically significant difference in rate of medication adherence (p=0.20) as assessed by pill count at midline and endline. The study-provided phone group exhibited higher medication at midline compared to the BYOD group as shown in Figure 1. However, they also displayed a directional trend (p=0.20) of greater decline in medication adherence than the BYOD group from the midline to endline assessment periods, where the study-provided phone group saw a 5.7% decline in medication adherence from midline to endline, and the BYOD group saw a 0.9% decline in medication adherence over the same period. Table 1 describes pill count ratios for both groups at midline and endline.

**Table 1:** Descriptive statistics for pill count ratio by study group and period

Group	Period						
	Midline			Endline			
	N	Range	Mean (SD)	N	Range	Mean (SD)	
Study-provided phone	26	0.58-0.93	0.81 (0.08)	29	0.19-1.00	0.76 (0.19)	
BYOD	27	0.19-1.00	0.76 (0.17)	28	0.50-1.00	0.75 (0.13)	

**Figure 2:**
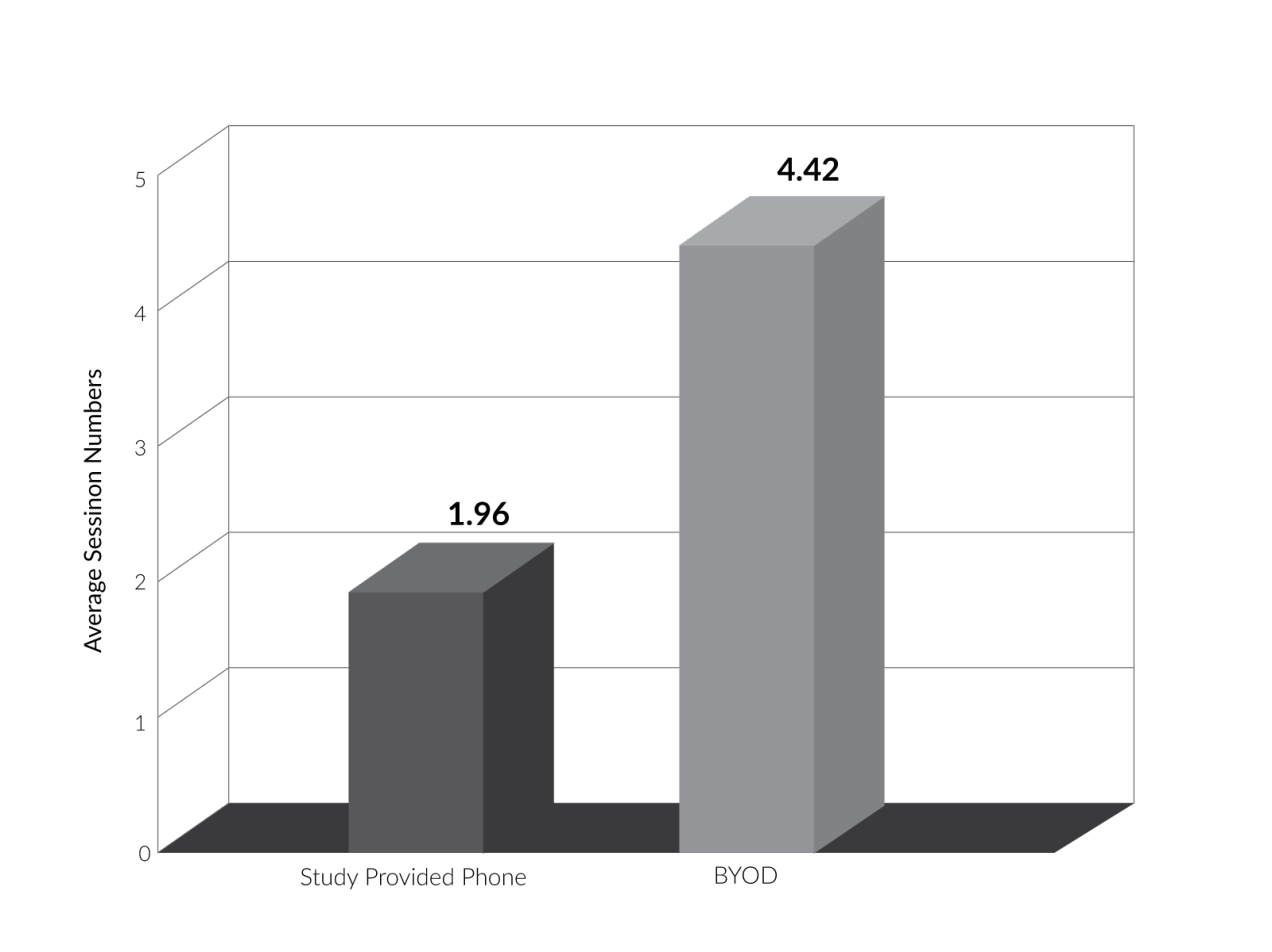
Average number of application sessions per day

**Figure 3:**
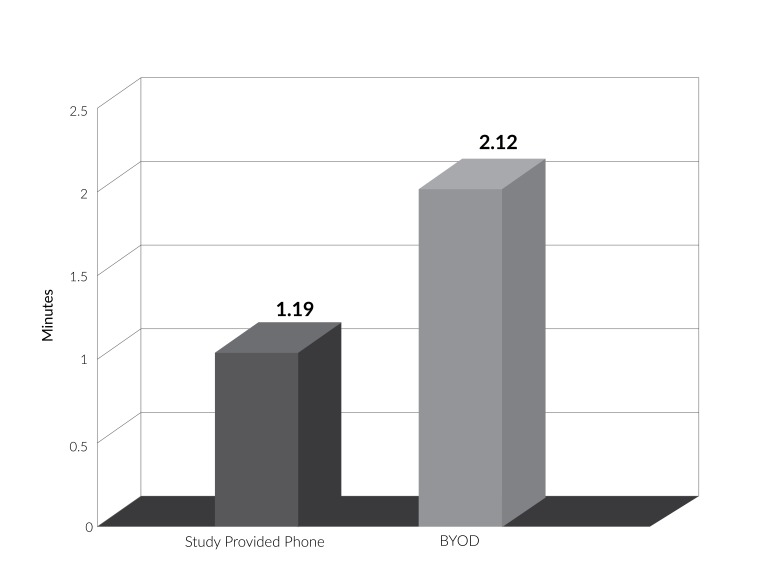
Average duration of application usage per day

The BYOD subgroup showed significantly higher engagement with the intervention, as measured by frequency and duration of application use over the study period, than did the study-provided phone subgroup, based on data collected from Google Analytics. The average number of application sessions per day, and concurrently the average time spent in the application per day, was significantly greater among BYOD users as compared to study-provided phone users (t(32)=3.52, p<0.001; t(40)=2.79, p=0.01 respectively). As seen in Figures 2-3, while the BYOD group had an average of 4.42 application sessions per day, translating to an average of 2.12 total minutes in the application per day, the study-provided phone users had an average of 1.96 application sessions per day, with an average of 1.19 total minutes in the application per day. Table 2 describes the frequency and duration of application usage for both groups over the study period.

**Table 2:** Descriptive statistics for frequency and duration of application usage by study group

Group	Average number of application sessions per day			Average duration of application usage per day (minutes)		
	Range	Mean (SD)	p value	Range	Mean (SD)	p value
Study-provided phone	0.10-5.75	1.96 (0.21)	<0.001	0.02-2.67	1.19 (0.15)	0.01
BYOD	0.22-15.82	4.42 (0.66)		0.19-6.53	2.12 (1.57)	

The BYOD subgroup demonstrated a significant effect of engagement on medication adherence in the endline period of the study for number of application sessions per day (beta=0.0006, p=0.02) and duration of application usage per day (beta=0.00001, p=0.03). This effect is not seen in the BYOD group at midline or in the study-provided phone group at midline and endline. Table 3 presents the square of the correlation coefficients for assessment of the relationships between application engagement and medication adherence. 

**Table 3:** Correlation coefficients for associations between application engagement and medication adherence

Group	Average number of application sessions per day predicting medication adherence				Average duration of application usage per day predicting medication adherence			
	Midline period		Endline period		Midline period		Endline period	
	R-squared	p value	R-squared	p value	R-squared	p value	R-squared	p value
Study-provided phone	0.0004	0.92	0.0934	0.11	0.0003	0.93	0.0364	0.34
BYOD	0.048	0.27	0.199	0.02	0.067	0.2	0.162	0.03

Based on qualitative interview data, all participants in the BYOD group reported that the BYOD model was highly usable and participants in both groups reported that, given the option of either model, they would prefer to use the application on their own device rather than a study provided device. BYOD users found it convenient to use their own phone as it allowed the mobile application to integrate easily into their lifestyle. This echoed reports from study-provided phone users who reported that it was inconvenient to carry a second device. Study-provided phone users disliked the additional responsibility of using a study phone, often mentioning that they were afraid of losing or breaking it. Most study-provided phone users, moreover, reported keeping their study phone at home, often near their bottle of study medication, instead of carrying the device with them throughout the day. This often meant that such users did not have the opportunity to interact with the application throughout the day, but only in the morning and evening when they were at home. With fewer opportunities to interface with the application, it is possible that being in the study-provided device subgroup would negatively impact a participant’s engagement in the study as a whole.

## Discussion

To our knowledge, this is the first randomized control trial (RCT) to assess the BYOD model in the context of a mobile patient engagement application in support of clinical research. Study findings indicate the high usability and feasibility of the BYOD method in promoting participant engagement with a mobile application and in reinforcing medication adherence compared to the current standards for study provisioning devices [6].The medication adherence rates between the two intervention sub-groups assessed indicate the comparable effectiveness of the BYOD and study-provided phone models in supporting medication adherence over the 28-day study period. While the study-provided phone users displayed greater medication adherence at midline, they also displayed a greater decline as the study period progressed, whereas BYOD users exhibited very little change in medication adherence over time. Due to the limited study length, this suggests that further studies should be implemented to see whether this trend persists over greater study durations and larger samples.

Duration and frequency of intervention use differed significantly across the two study groups. The study-provided phone group opened the application just roughly twice per day—the same number of times they were asked to log their study medication intake per the study protocol. This supports qualitative findings that study-provided device users often left their study iPhone at home, accessing the application only in the morning and in the evening when they were at home, whereas the BYOD users accessed the application regularly and repeatedly throughout the day, leading to longer durations of time spent in the mobile application per day.

The BYOD group demonstrated a significant effect of engagement on medication adherence in the endline period of the study, though not at midline, indicating that greater engagement with the mobile application correlates to greater medication adherence in later periods of the study. This finding should be interpreted as a directional trend strongly suggesting that mobile application engagement may affect medication adherence in later periods of the study in the BYOD group. Due to the small size of the BYOD subgroup in the study design, there is not sufficient power to assess the significance of this trend with full statistical rigor; notably, confounders to medication adherence may also produce this trend. Moreover, it is important to recognize that results may be affected by the communication content, by individual participant motivational factors, and by the software itself; the assessed strategies and findings must be interpreted in the context of our target population and study setting. Additionally, the lack of allocation concealment or participant blinding may have introduced bias, and further cross-platform validation work will be necessary to ensure that BYOD does not introduce any unnecessary bias in collected data.

Methodological limitations notwithstanding, findings provide important preliminary evidence of a correlation between application engagement and medication adherence in later periods of the study particularly within the BYOD group. The importance of this outcome for the conduct of clinical research cannot be overemphasized; strategies to improve adherence to study protocol and reduce attrition have the potential to increase power and generalizability of results, while the BYOD models presents an opportunity to do so while also reducing costs and enhancing participant satisfaction [11]. The potential gains are especially marked in late-phase studies.

## Conclusions

These results provide early and novel evidence that the BYOD model can be feasible and usable in health research settings, and that it contributes to a better participant experience. Participants reported a strong preference for the BYOD model of delivery and were found to access the application significantly more frequently and for greater durations when using the application, supporting the hypothesis that the BYOD is the more usable model. While there was no significant difference in medication adherence between the BYOD and study-provided phone users, the trend of decline in medication adherence observed in the study-provided phone users supports the need to replicate findings over a longer study period to test whether the BYOD model may be more effective in sustaining engagement and medication adherence over time. The significant effect of application engagement on medication adherence in the BYOD subgroups indicates that greater engagement with the application correlates to greater medication adherence. Further research, incorporating larger sample sizes, is also warranted to see if these findings can be replicated in longer study durations and among differing populations.
